# Persistent Intestinal Obstruction After Reduction of Incarcerated Inguinal Hernia: A Case of "Reduction en Masse"

**DOI:** 10.7759/cureus.94340

**Published:** 2025-10-11

**Authors:** Muthanna Al-Jumaili, Mohammed Alshawi, Mojib Fallah, Tina A Soerensen, Issam Al-Najami

**Affiliations:** 1 Department of Surgery, Odense University Hospital, Odense, DNK; 2 Department of Surgery, Kolding Hospital, Kolding, DNK

**Keywords:** bowel incarceration, hernia reduction, inguinal hernia, intestinal obstruction, reduction en masse

## Abstract

"Reduction en masse" is a rare condition in which a hernia can be reduced to the abdomen, but the bowel remains trapped in the hernia sac. Although this is a rare condition, a delayed diagnosis can lead to serious and life-threatening consequences. In this case report, a 60-year-old woman presented with clinical symptoms of small bowel obstruction at the emergency department, who later developed recurrent symptoms of small bowel obstruction after seemingly successful reduction of a right-sided inguinal hernia. This led to diagnostic laparoscopy, where a small bowel segment trapped in a hernia sac was found.

## Introduction

"Reduction en masse" is a rare clinical diagnosis in which an inguinal hernia, including the hernia sac, is reduced into the abdominal cavity, giving an impression of successful reduction externally. However, the incarcerated bowel remains trapped within the peritoneal defect at the neck of the hernia sac [1]. It most commonly occurs following a forceful reduction of a hernia, but it may also arise spontaneously [1-3].

The incidence of "reduction en masse" is estimated to be approximately 1 in 13,000 cases of inguinal hernia [4]. Although it is relatively uncommon, a missed diagnosis may lead to serious complications. Reduction of the hernia through the abdominal wall defect is ineffective, as the condition can persist as an internal hernia [5].

Early diagnosis remains challenging due to its rarity and nonspecific clinical presentation. In some cases, a definitive diagnosis may require advanced imaging, such as computed tomography (CT), or even diagnostic laparoscopy [4,5]. A delay in the detection of this complication may result in ischemia and subsequent necrosis of the incarcerated intestinal segment [4].

Although it can be diagnosed based on a specific CT finding, many radiologists may not recognize it, as it represents a very rare complication. A distinctive radiological feature known as the "preperitoneal hernia sac sign" has been described, in which the hernia sac and its contents are visualized within the preperitoneal space, adjacent to the inguinal fossa [6].

In this report, we describe the case of a patient with persistent intestinal obstruction after manual reduction of an inguinal hernia.

## Case presentation

A 60-year-old woman with no significant medical history, known to have an asymptomatic right-sided inguinal hernia, presented to the emergency department with gradually worsening diffuse abdominal pain throughout the day.

On physical examination, a 5x5 cm tender and tense mass was palpated in the right groin with normal overlying skin. Blood tests revealed a mild leukocytosis and normal C-reactive protein (CRP).

Manual reduction of the hernia into the abdominal cavity was performed under morphine sedation, resulting in relief of the patient's pain. After 5 hours of observation, the patient was discharged with a plan for elective hernia repair.

The patient was subsequently readmitted with nonspecific abdominal pain, occasional vomiting, and absence of bowel movements. On examination, there was diffuse abdominal tenderness without signs of local or generalized peritonitis. The hernia was no longer visible or palpable, and the condition was considered constipation. The patient was discharged again.

The patient returned to the emergency department with the same symptoms four days after the initial hernia reduction. This time, CRP was elevated at 82 mg/L, prompting a CT scan of the abdomen to rule out complications.

**Figure 1:**
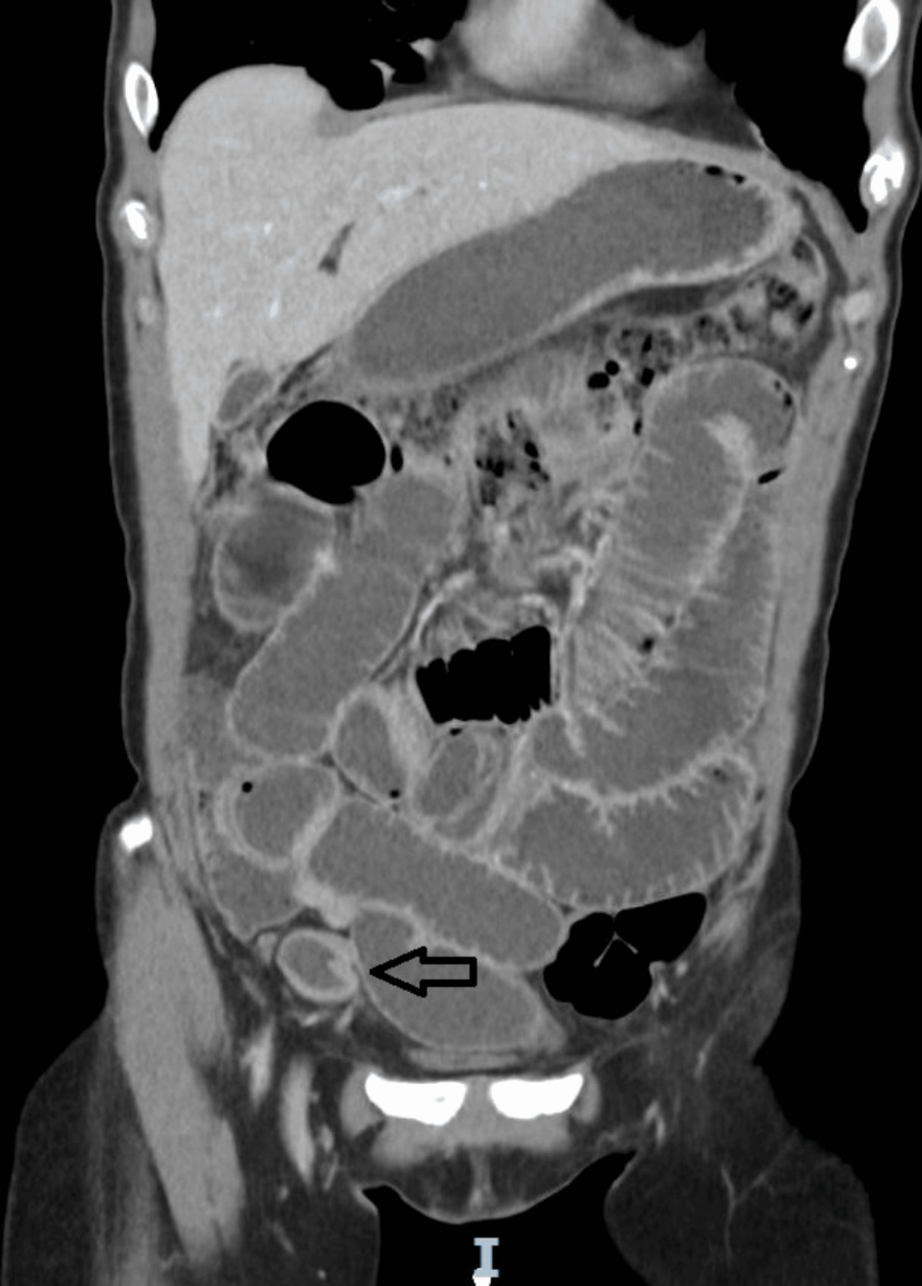
CT image showing that the patient had mechanical intestinal obstruction

The CT scan demonstrated mechanical small bowel obstruction with focal bowel wall thickening of a small intestinal segment near the abdominal wall defect (Figure 1).

**Figure 2:**
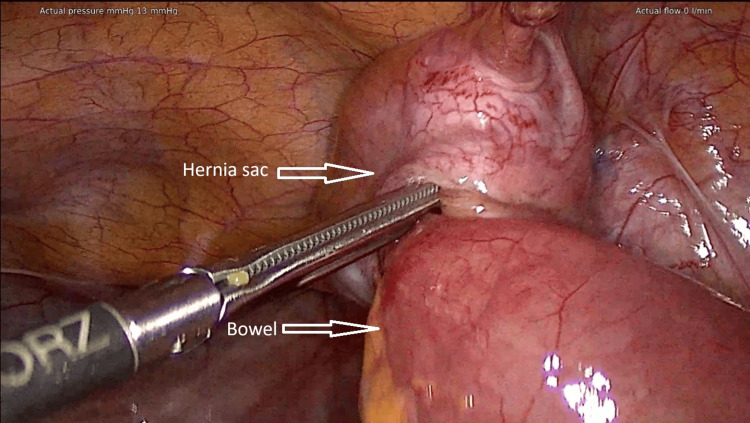
The image shows an incarcerated small bowel segment within a peritoneal defect (hernia sac), where both structures were objectively reduced into the abdominal cavity

A diagnostic laparoscopy was performed, revealing a "reduction en masse" with an incarcerated small bowel segment within a hernia sac that had been reduced intra-abdominally. The bowel was successfully freed from the hernia sac laparoscopically without the need for bowel resection (Figure 2). The peritoneal defect was closed using non-absorbable sutures.

The patient was discharged two days later with a plan for elective definitive surgical repair of the hernia.

## Discussion

Mechanical small bowel obstruction is among the most common acute surgical conditions seen in the emergency department [7]. "Reduction en masse" is a rarely described cause of small bowel obstruction [4,5,8]. If unrecognized, this condition may have serious consequences.

Similar cases have been reported as reduction en masse, either occurring spontaneously or following manual reduction by a physician [1-6,8]. However, in our case, the patient continued to experience persistent symptoms of intestinal obstruction following manual reduction of an inguinal hernia. Notably, these symptoms led to multiple hospital readmissions, despite the absence of any overt hernia-related complaints thereafter.

The diagnosis should be suspected in cases of recurrent or persistent ileus symptoms following hernia reduction, even in the absence of a visible or palpable hernia.

The exact pathogenesis remains unclear, but it is thought that prolonged asymptomatic incarceration leads to fibrotic adhesions between the bowel and the hernia sac, preventing the bowel from freely exiting the sac even after apparent reduction [8].

Diagnosing the condition is challenging, as the clinical signs present before reduction may no longer be present. A significant risk factor identified in previous case reports is the presence of a chronically reducible hernia, often associated with increasing difficulty during reduction due to fibrosis from repeated attempts [5].

The cornerstone of diagnosis is a thorough patient history and physical examination, indicating persistent signs of bowel obstruction despite apparent hernia reduction [8]. Abdominal CT imaging should be prioritized to establish the diagnosis and guide urgent management [5]. In this case, the patient underwent laparoscopic surgery for the acute management of the obstruction and to assess bowel viability.

In the acute setting, closure of the peritoneal defect may be considered to prevent recurrent incarceration. Definitive herniotomy was deferred in this case to allow resolution of inflammation prior to mesh placement, thereby reducing the risk of infection and complications [5].

## Conclusions

"Reduction en masse" is a rare but clinically significant cause of small bowel obstruction. This case emphasizes the importance of considering this diagnosis in patients with persistent or recurrent abdominal pain following inguinal hernia reduction, particularly in those with a long-standing history of herniation and increasing difficulty with reduction. Delayed or missed diagnosis may lead to bowel strangulation.

A high index of suspicion is essential in patients presenting with persistent or recurrent obstructive symptoms following hernia reduction. Abdominal CT imaging plays a crucial role in identifying this condition and guiding timely surgical intervention. Early recognition and surgical intervention, preferably laparoscopic, can prevent bowel ischemia and optimize patient outcomes.

Awareness of this rare entity among clinicians and radiologists is imperative to reduce diagnostic delays and associated morbidity.
